# Hemodynamic Outcomes of Anesthesia Management Assisted by Nurses Trained in Specific Medical Acts in Gastrointestinal Surgery: A Retrospective Propensity Score-Matched Pilot Study

**DOI:** 10.7759/cureus.111251

**Published:** 2026-06-21

**Authors:** Norito Kumagai, Tomonori Sugawara

**Affiliations:** 1 Anesthesiology, Takamatsu Red Cross Hospital, Takamatsu, JPN

**Keywords:** anesthesia management, intraoperative hypotension, propensity score matching, specific act training nurse, task-shifting

## Abstract

Introduction

Task-shifting in anesthesia care is increasingly recognized as a vital approach to managing anesthesiologist shortages. In Japan, a national certification system for Nurses Trained in Specific Medical Acts (NTSMAs) was introduced to support perioperative management. This pilot study aimed to compare intraoperative hemodynamic outcomes between NTSMA-assisted anesthesia management and physician-only management in gastrointestinal surgery using a propensity score-matched design.

Materials and methods

We retrospectively reviewed patients who underwent elective gastrointestinal surgery under general anesthesia between 2023 and 2026. A 1:1 propensity score matching (PSM) was conducted using age, sex, American Society of Anesthesiologists Physical Status (ASA-PS), and surrogate markers of anticipated procedural complexity (anesthesia time and surgical time) to balance baseline characteristics between the NTSMA-assisted group and the physician-only control group. The primary outcome was the incidence of intraoperative hypotensive screening events, defined as systolic blood pressure <80 mmHg at least once. Secondary outcomes included total intraoperative doses of vasopressors, including phenylephrine, ephedrine, and norepinephrine.

Results

After 1:1 matching, 99 pairs (total N = 198) were analyzed, with all covariates achieving a standardized mean difference of <0.1. The incidence of intraoperative hypotensive screening events was not significantly different between the NTSMA-assisted and physician-only groups (67.7% vs. 64.6%). Conditional logistic regression analysis demonstrated no statistically significant association between NTSMA involvement and hypotensive events (OR: 1.150, 95% CI: 0.632 to 2.094, p = 0.648). Additionally, no significant differences were observed in total intraoperative requirements, evaluated using the Wilcoxon signed-rank test, for phenylephrine (p = 0.385), norepinephrine (p = 0.930), or ephedrine (p = 0.069).

Conclusions

In this retrospective pilot study, NTSMA-assisted anesthesia management did not show a statistically significant difference in the incidence of intraoperative severe hypotension compared with physician-only management. However, given the limited sample size and the highly sensitive nature of our primary outcome, these findings should be considered strictly hypothesis-generating. Larger, prospectively designed non-inferiority trials are required to definitively establish the clinical safety and non-inferiority of NTSMA-assisted task-shifting in anesthesia care.

## Introduction

The absolute shortage and unequal distribution of anesthesiologists, combined with their escalating workloads, represent critical challenges for healthcare systems globally. To optimize resource utilization and maintain the quality of perioperative care, task-shifting models involving non-physician anesthesia providers, such as Certified Registered Nurse Anesthetists (CRNAs), have been widely implemented in Western nations [1]. Extensive large-scale clinical evidence has established that anesthesia care models incorporating supervised nurse anesthetists do not compromise patient safety or worsen postoperative outcomes. In addition, task-shifting and task-sharing in surgical and anesthesia care have been discussed internationally as strategies to address workforce shortages and maintain access to essential perioperative services [2,3].

In Japan, to facilitate team-based medicine and address physician overwork, a national certification framework designated as “Nurses Trained in Specific Medical Acts” (NTSMAs) was legally enacted. Under this framework, nurses who successfully complete standardized training packages, including the intraoperative anesthesia management domain, are authorized to perform specific medical procedures under the indirect supervision of a designated anesthesiologist. These procedures encompass the fine-tuning of mechanical ventilator settings, adjustment of IV fluid rates, and administration of temporary vasoactive medications in response to blood pressure fluctuations within strict, pre-agreed protocols, with a supervising anesthesiologist immediately available for direct intervention. A recent narrative review of anesthesia assistance in Japan described nurses as a major group of anesthesia assistants and reported that several educational pathways, including specified medical acts training, have been developed to support team-based anesthesia care [4].

Despite the institutional expansion of this certification, empirical data quantitatively validating the safety and clinical feasibility of NTSMA-assisted anesthesia care within Japanese clinical practice remain extremely scarce. Managing intraoperative hemodynamics is a fundamental task, as even brief periods of intraoperative hypotension are independently associated with an elevated risk of postoperative acute kidney injury and myocardial injury [5]. Therefore, this retrospective, propensity score-matched pilot study aimed to evaluate the preliminary feasibility of intraoperative hemodynamic management under NTSMA assistance by comparing it with traditional physician-only management in patients undergoing gastrointestinal surgery.

## Materials and methods

Study design and institutional review

This study was a retrospective, single-center, observational pilot study conducted at Takamatsu Red Cross Hospital, Japan. The study protocol was approved by the Institutional Review Board (IRB) of Takamatsu Red Cross Hospital (26-005). Due to the retrospective nature of the study, which used historical, de-identified clinical data obtained from routine medical care, the requirement for written informed consent was waived by the IRB. Information regarding the study objectives and data usage was publicly disclosed via an institutional opt-out process on the hospital website, ensuring that patients had the opportunity to refuse data inclusion. The study reporting adheres to the Strengthening the Reporting of Observational Studies in Epidemiology (STROBE) guidelines [6]. A convenience sampling approach was adopted based on the available data within the study period, and no formal prior sample size calculation was performed.

Patient population and data source

Clinical data were retrieved from the institutional electronic anesthesia information management system (PrescientOR®, Fujifilm Medical, Japan). All potentially eligible cases were identified according to procedure category, anesthesia type, and surgery date. We focused on adult patients who underwent elective laparoscopic or robot-assisted gastrointestinal surgeries under general anesthesia between January 2023 and March 2026 because this was the clinical setting in which NTSMA-assisted anesthesia management had been implemented at our institution, and limiting the cohort to this surgical category reduced heterogeneity in operative invasiveness and perioperative workflow. Cases outside this predefined target population, including non-gastrointestinal surgery, emergency surgery, pediatric cases, or records lacking the required anesthesia or medication data, were not included in the analysis. Extracted variables included demographic characteristics, American Society of Anesthesiologists Physical Status (ASA-PS) classification, anesthesia time, surgical time, intraoperative blood pressure records, and total administered doses of vasopressors.

No upper age limit was applied. Elderly patients were included because they represent a clinically important population in gastrointestinal surgery, and age was incorporated into the propensity score model to account for age-related differences in baseline risk. No pregnant patients were included in the analyzed cohort. A complete-case analysis was used to handle missing values, which were minimal across the target variables.

Exposure and variables

The study cohort was divided into two groups based on the intraoperative care provider team structure: the NTSMA-assisted group and the physician-only group. The NTSMA-assisted group comprised cases in which the certified NTSMA was continuously stationed at the bedside and primarily adjusted anesthetic agents, IV fluids, and vasopressors based on strict, pre-agreed protocols, with a supervising anesthesiologist immediately available for direct intervention (indirect supervision). The NTSMA completed the national training package for intraoperative anesthesia management between 2022 and 2023 and began clinical implementation in 2023. Therefore, the NTSMA-assisted cases in this study reflect anesthesia management during the early phase after implementation of this role at our institution. The physician-only group included cases managed exclusively by board-certified anesthesiologists or anesthesia residents under direct supervision. Residents, when involved, provided anesthesia care only under the direct supervision of board-certified anesthesiologists. However, the exact proportion of resident-involved cases, the postgraduate year of each resident, and individual provider-level experience could not be reliably extracted from the electronic database, which represents a potential source of residual confounding.

The baseline covariates extracted for matching included age, sex, and American Society of Anesthesiologists Physical Status (ASA-PS) classification [7]. Additionally, to account for exposure-independent baseline difficulty, total anesthesia time (minutes) and total surgical time (minutes) were used as surrogate markers of anticipated procedural complexity in the matching model, acknowledging that these parameters may be post-exposure and therefore require cautious epidemiological interpretation. Both total IV anesthesia and volatile anesthesia were used in routine clinical practice in the study population. Detailed anesthetic techniques, including the use of total IV anesthesia versus volatile anesthesia and the exact multimodal analgesic regimens, were not incorporated into the propensity score model because these provider- and case-level details could not be comprehensively extracted in a standardized manner.

Outcomes

The primary outcome was defined as the occurrence of an intraoperative hypotensive screening event. Although intraoperative hypotension has been defined using various thresholds, including mean arterial pressure <65 mmHg, systolic blood pressure (SBP) <80 mmHg, or a relative decrease of more than 20%-30% from baseline blood pressure, we deliberately adopted a single absolute SBP threshold for this retrospective pilot study. This decision was made because SBP <80 mmHg is a commonly used absolute threshold in observational research [5], and because the retrospective electronic anesthesia database allowed more reliable extraction of the occurrence of an absolute SBP threshold than calculation of time-weighted or baseline-relative blood pressure reductions. To prioritize patient safety and detect even transient hemodynamic fluctuations during NTSMA-assisted anesthesia, we defined the primary outcome as any SBP drop below 80 mmHg, regardless of its duration. Because this threshold captures any single brief episode, it may include transient decreases immediately following anesthetic induction rather than reflecting sustained hemodynamic instability. Therefore, this outcome should be interpreted as a highly sensitive screening metric for intraoperative hypotension rather than a definitive marker of sustained circulatory failure or clinically relevant hypotension. Secondary outcomes included the total intraoperative doses of standard vasopressors administered, specifically phenylephrine (mg), ephedrine (mg), and norepinephrine (mg).

Statistical analysis

To mitigate potential selection bias and confounding inherent in retrospective observational studies, such as the tendency to assign NTSMAs to younger or healthier patients, we performed 1:1 propensity score matching (PSM) [8]. Propensity scores for the likelihood of receiving NTSMA-assisted management were calculated using a logistic regression model incorporating age, sex, ASA-PS, anesthesia time, and surgical time. Matching was performed using the nearest-neighbor method without replacement. Standardized mean differences (SMDs) were calculated to evaluate covariate balance, with an SMD <0.1 indicating acceptable balance between the two matched groups.

Following matching, statistical analyses for paired data were strictly applied to respect the paired structure of the matched data. For the binary categorical primary outcome, incidence of hypotensive screening events, conditional logistic regression analysis was conducted to account for the stratified pair structure, and the OR with its 95% CI was calculated. Continuous variables representing total vasopressor doses typically exhibit skewed distributions with a high frequency of zero values; thus, they were compared using the Wilcoxon signed-rank test and summarized using medians with IQRs alongside means ± SDs. All statistical analyses were conducted using Python (v3.9), with a two-tailed p-value <0.05 considered statistically significant. To address potential collider bias and overmatching introduced by including post-exposure variables, namely anesthesia and surgical times, a sensitivity analysis was conducted. In this sensitivity analysis, the propensity score model was strictly restricted to preoperative baseline characteristics, including age, sex, and ASA-PS.

## Results

The initial unmatched database comprised 99 patients in the NTSMA-assisted group and 191 patients in the physician-only control group. Prior to matching, substantial baseline imbalances were present: the physician-only group was significantly younger (mean age: 65.0 ± 15.4 vs. 71.8 ± 10.2 years, SMD = 0.429) and had lower physical status risk (mean ASA-PS: 2.1 ± 0.7 vs. 2.4 ± 0.6, SMD = 0.386) compared with the NTSMA-assisted group.

Following 1:1 propensity score matching, 99 well-balanced pairs (total N = 198) were generated. Post-matching, all baseline covariates achieved excellent balance, demonstrated by SMDs well below the 0.1 threshold (Table 1).

**Table 1:** Patient baseline characteristics before and after propensity score matching. ASA-PS: American Society of Anesthesiologists Physical Status; SMD: Standardized mean difference; NTSMA: Nurse Trained in Specific Medical Acts.

Variable	Pre-matching: NTSMA-assisted group (N = 99)	Pre-matching: control group (N = 191)	Pre-matching SMD	Post-matching: NTSMA-assisted group (N = 99)	Post-matching: control group (N = 99)	Post-matching SMD
Age (years), mean ± SD	71.8 ± 10.2	65.0 ± 15.4	0.429	71.8 ± 10.2	71.7 ± 11.5	0.009
Male, n (%)	62 (62.6%)	110 (57.6%)	0.102	62 (62.6%)	64 (64.6%)	0.041
ASA-PS, mean ± SD	2.4 ± 0.6	2.1 ± 0.7	0.386	2.4 ± 0.6	2.4 ± 0.7	0
Anesthesia time (min), mean ± SD	285.3 ± 132.4	258.1 ± 140.2	0.199	285.3 ± 132.4	290.5 ± 128.6	0.04
Surgical time (min), mean ± SD	238.6 ± 125.8	215.4 ± 130.5	0.181	238.6 ± 125.8	242.1 ± 121.3	0.028

In the matched cohort, hemodynamic outcomes showed no statistically significant differences between the two groups (Table 2). The incidence of intraoperative hypotensive screening events (SBP <80 mmHg) was 67.7% (67/99 cases) in the NTSMA-assisted group and 64.6% (64/99 cases) in the physician-only group. Conditional logistic regression analysis revealed that NTSMA involvement was not associated with a statistically significant increase in hypotensive screening events (OR: 1.150, 95% CI: 0.632 to 2.094, p = 0.648). Furthermore, the sensitivity analysis restricted to purely preoperative variables successfully yielded a balanced cohort of 95 matched pairs (all SMDs <0.1). Consistent with the primary analysis, the incidence of intraoperative severe hypotension did not differ significantly between the NTSMA-assisted group and the physician-only group (68.4% vs. 70.5%; OR: 0.905, 95% CI: 0.488-1.680, p = 0.753), confirming the robustness of our findings.

**Table 2:** Comparison of intraoperative outcomes in the matched cohort. ᵃ Calculated using conditional logistic regression analysis to account for matched-pair strata. ᵇ Evaluated using the Wilcoxon signed-rank test to account for skewed distributions with a high frequency of zero values. NTSMA: Nurse Trained in Specific Medical Acts.

Outcome	NTSMA-assisted group (N = 99)	Control group (N = 99)	Statistical metric	p-value
Intraoperative hypotensive screening event, n (%)	67 (67.7%)	64 (64.6%)	Odds ratio: 1.150 (95% CI: 0.632 to 2.094)ᵃ	0.648
Total phenylephrine dose (mg)	Median (IQR): 0.10 (0.00-0.45) (mean ± SD: 0.28 ± 0.40)	Median (IQR): 0.00 (0.00-0.30) (mean ± SD: 0.35 ± 0.71)	Non-parametric analysisᵇ	0.385
Total norepinephrine dose (mg)	Median (IQR): 0.00 (0.00-0.00) (mean ± SD: 0.08 ± 0.23)	Median (IQR): 0.00 (0.00-0.00) (mean ± SD: 0.08 ± 0.38)	Non-parametric analysisᵇ	0.93
Total ephedrine dose (mg)	Median (IQR): 4.00 (0.00-12.00) (mean ± SD: 6.69 ± 6.57)	Median (IQR): 8.00 (0.00-14.00) (mean ± SD: 8.83 ± 8.61)	Non-parametric analysisᵇ	0.069

Regarding secondary outcomes, the total intraoperative requirements for vasopressors were statistically comparable between the groups. There were no significant differences in the total doses of phenylephrine (Wilcoxon test, p = 0.385) or norepinephrine (Wilcoxon test, p = 0.930). The intraoperative ephedrine dose showed a lower numerical pattern in the NTSMA-assisted group than in the physician-only group, although this difference did not reach conventional statistical significance in the non-parametric exploratory analysis (Wilcoxon test, p = 0.069).

## Discussion

This single-center retrospective matched cohort study evaluated the preliminary hemodynamic feasibility of intraoperative anesthesia management assisted by certified NTSMAs in Japan using a structured propensity score-matched approach. By implementing paired statistical analyses and conditional logistic regression after matching, our findings demonstrate that NTSMA involvement in the anesthesia care team was not associated with statistically significant worsening of the measured hemodynamic outcomes during elective gastrointestinal surgeries.

The implementation of PSM was intended to counter potential institutional selection bias. In observational evaluations of anesthesia staffing models, assignment patterns may favor healthier or lower-risk patients, creating potential treatment-selection bias [9]. Our matching process effectively countered this bias, creating balanced cohorts with a mean patient age exceeding 70 years and a mean anesthesia duration approaching five hours. Because geriatric patients may be more susceptible to physiologic changes associated with pneumoperitoneum, positional changes, and anesthetic-induced cardiovascular depression, the absence of an upper age cut-off and the inclusion of age in the propensity score model are important aspects of the present study design. Even within this older demographic undergoing invasive gastrointestinal procedures, hemodynamic outcomes did not demonstrate major deterioration compared with the traditional physician-only care model.

Regarding the secondary outcomes, the total dose of ephedrine showed an exploratory numerical trend toward a lower requirement in the NTSMA-assisted group, although it did not achieve statistical significance (p = 0.069). This finding should be interpreted cautiously because the pilot study was not powered to detect differences in individual vasopressor use. We can only cautiously hypothesize that the constant presence of an NTSMA at the patient’s bedside may facilitate close visual assessment, potentially allowing for preemptive micro-adjustments within the protocol, such as fluid infusion adjustments or anesthetic depth corrections, before reactive rescue boluses of ephedrine are needed. However, further prospective evaluation is required to confirm this mechanism.

Our findings are broadly consistent with previous observational studies suggesting that anesthesia care team models involving non-physician anesthesia providers or anesthesia nurses may be implemented without clear worsening of perioperative outcomes when appropriate physician involvement is maintained [10,11]. For example, Sun EC et al. reported no significant differences in mortality, length of stay, or inpatient spending according to anesthesia care team composition, while Cordovani D et al. reported that the presence of an anesthesia nurse in a supervised anesthesia care team was associated with lower 30-day mortality and shorter hospital stay compared with solo anesthesiologist care [10,11]. Although these studies were conducted in healthcare systems that differ substantially from Japan and are not directly comparable with NTSMA-assisted anesthesia management, they support the concept that the structure of the anesthesia care team itself is an important factor in perioperative outcomes.

Importantly, our results should not be interpreted as supporting independent anesthesia practice by NTSMAs. In our institutional model, the NTSMA remained at the bedside and performed protocol-based adjustments, while a supervising anesthesiologist was immediately available for direct intervention. This distinction is clinically important because recent evidence suggests that anesthesiologist staffing ratios and overlapping clinical responsibilities may be associated with patient outcomes in anesthesia care team models [9]. Therefore, the apparent hemodynamic feasibility observed in the present study may depend not only on the presence of an NTSMA but also on the availability of clear protocols, defined escalation criteria, and close physician supervision.

The high incidence of hypotensive screening events in both groups should also be interpreted cautiously. Although intraoperative hypotension has been associated with postoperative organ injury in observational studies [5], a recent meta-analysis of randomized trials suggests that the relationship between intraoperative blood pressure targets and postoperative outcomes remains complex [12]. In the present study, the primary outcome was deliberately defined as any single SBP decrease below 80 mmHg to prioritize sensitivity. Therefore, the absence of a significant difference between groups should be understood as a preliminary signal that NTSMA involvement did not increase transient hypotensive screening events, rather than as evidence of equivalent postoperative organ protection.

We acknowledge several limitations in this study. First, it was a retrospective analysis conducted at a single institution. Furthermore, this pilot study was inherently limited by its relatively small sample size, which restricted our statistical power. The wide 95% CI for the primary outcome indicates that we cannot entirely rule out the possibility of a Type II error. Therefore, the lack of a statistically significant difference should not be misinterpreted as definitive proof of clinical equivalence or non-inferiority between the two groups. Second, due to data extraction constraints from the electronic database, intraoperative hypotension was defined using a simplified absolute threshold (any single SBP <80 mmHg). The relatively high incidence of the primary outcome in both groups, over 60%, is a direct reflection of our rigorous, safety-first methodology. By using a highly sensitive screening metric that captured even transient, self-correcting blood pressure drops frequently observed immediately after anesthesia induction, we aimed to ensure that no potential hemodynamic instability associated with task-shifting was overlooked. Consequently, this screening metric does not necessarily reflect sustained hemodynamic collapse, postoperative acute kidney injury, or inadequate clinical care. Third, unmeasured residual confounding factors, such as preoperative patient comorbidities (e.g., chronic hypertension and cardiac dysfunction), chronic antihypertensive medication use (e.g., angiotensin-converting enzyme (ACE) inhibitors and beta-blockers), surgical approach (laparoscopic vs. robotic), intraoperative blood loss, individual provider experience, resident postgraduate year, and detailed anesthetic techniques, such as total intravenous anesthesia versus volatile anesthesia and multimodal analgesic strategies, could not be fully adjusted. These factors may influence intraoperative blood pressure management and vasopressor requirements. Fourth, while including anesthesia and surgical times as surrogate markers for procedural complexity introduces a theoretical risk of post-exposure bias, our sensitivity analysis, restricted entirely to strictly preoperative variables, yielded highly consistent results. This underscores the robustness of our conclusion despite the retrospective nature of the study.

Despite these limitations, as a hypothesis-generating pilot study, this report provides preliminary data supporting the institutional feasibility of task-shifting in Japanese anesthesia care. These findings should be interpreted in the context of previous literature indicating that anesthesia-related task-shifting remains less extensively studied than surgical task-shifting and that available findings remain heterogeneous [3]. Utilizing NTSMAs within a structured anesthesia care team model under clear physician supervision can support workflow without showing signs of acute hemodynamic harm, providing a practical framework to alleviate the clinical burden on anesthesiologists.

## Conclusions

In this retrospective pilot study, NTSMA-assisted anesthesia management did not show a statistically significant difference in the incidence of intraoperative severe hypotension compared with physician-only management. However, given the limited sample size and the highly sensitive nature of our primary outcome, these findings should be considered strictly hypothesis-generating. Larger, prospectively designed non-inferiority trials are required to definitively establish the clinical safety and non-inferiority of NTSMA-assisted task-shifting in anesthesia care.
